# Proteomics analysis reveals the differential impact of the p97 inhibitor CB-5083 on protein levels in various cellular compartments of the HL-60 cell line

**DOI:** 10.17912/micropub.biology.001372

**Published:** 2024-11-27

**Authors:** Wenxuan Huang, Yanping Qiu, Diana Huynh, Ting-Yu Wang, Tsui-Fen Chou

**Affiliations:** 1 Biology and Biological Engineering, California Institute of Technology, Pasadena, California, United States; 2 Proteome Exploration Laboratory, Beckman Institute, California Institute of Technology, Pasadena, California, United States

## Abstract

Human p97/VCP is a vital AAA ATPase (ATPase associated with diverse cellular activity) that plays critical roles in protein homeostasis by regulating autophagy, endosomal trafficking, and the ubiquitin-proteasome system. Global proteomics analysis of p97/VCP inhibition with CB-5083 has been performed in HCT116 colon cells. Here, we examined the impact of CB-5083 treatment in another cancer model, the HL-60 acute myeloid leukemia cell line, employing subcellular fractionation combined with label-free proteomics to analyze changes in protein levels across cytoplasmic, nuclear, and insoluble membrane protein compartments. The results reveal distinct compartment-specific protein regulation, providing insight into p97/VCP’s cellular mechanisms and its potential for targeted therapeutic applications.

**Figure 1. Differential Impact of p97 Inhibitor CB-5083 on Protein Levels in HL-60 Cells.**
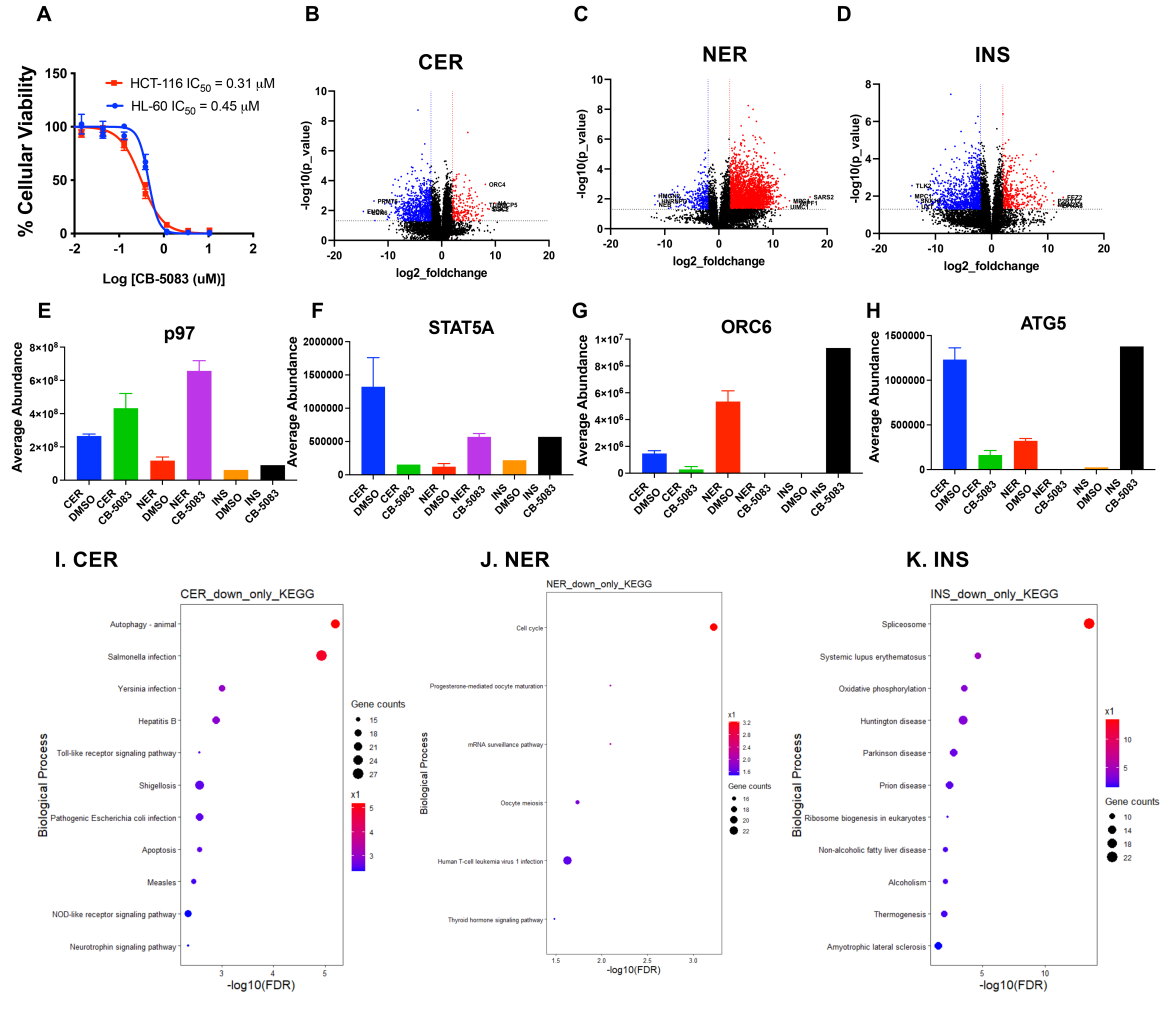
(A) Titration curves of CB-5083 in HCT116 and HL-60 cell lines. Cellular viability was determined after 48 hours.(B-D) Volcano plots comparing protein expression across different compartments relative to the DMSO control group are presented. The colors indicate the following: red represents upregulated proteins, blue denotes downregulated proteins, and black indicates proteins with no significant change compared to the DMSO control. (E-H) Protein abundance levels for several proteins discussed in the text are shown. Proteins without error bars indicate that only a single abundance value was determined.(I-K) Pathway analysis of significantly downregulated proteins in CER (I), NER(J), and INS(K).

## Description

**Introduction**

Valosin-containing protein (VCP/p97) is a highly conserved ATPase found across various species, where it plays multiple roles in cellular processes such as endosomal trafficking, autophagy, and mitochondria-associated degradation (Chou & Deshaies, 2011; Meyer et al., 2012) . Its central function in the ubiquitin-proteasome system (UPS) helps maintain protein homeostasis, making it a promising target for therapies against cancer and neurodegenerative diseases (Kilgas & Ramadan, 2023; Zhang et al., 2021; Boom & Meyer, 2017; Yu, 2024) . The first p97 active site inhibitor, DBeQ was discovered via a high-throughput screen, and subsequent structure-activity study identify ML240 and ML241 as improved p97 inhibitors (Chou, 2011, 2013) . These academic efforts provided an excellent starting point for Cleave Bioscience to develop CB-5038, the first-in-class ATP-competitive inhibitor of p97, which disrupts the UPS and endoplasmic reticulum-associated degradation (ERAD) pathways (Zhou, 2015) . Preclinical studies have shown its effectiveness against various cancers, including multiple myeloma (Le Moigne et al., 2017; Wang et al., 2021) . However, CB-5083 was withdrawn from clinical development after a Phase 1 trial due to off-target effects (Leinonen et al., 2024; Bastola & Chien, 2018) . The newer analogue CB-5339, without the off-target effect has completed a phase one clinical trial for acute myeloid leukemia (AML) (NCT04402541) based on the genetic evidence of AML dependence on p97/VCP (Roux, 2021) . We have also completed a clinical trial of CB-5339 in tumor-bearing dogs (LeBlanc 2022) . Previously, we compared proteomic changes in HCT116 colon cancer cells following treatment with CB-5083, assessing alterations in the global proteome (Wang, 2021) . To gain a deeper understanding of CB-5083's effects on global proteome changes in AML cancer cell, we used the HL-60 cell line—a well-established acute myeloid leukemia model which is also known for its sensitivity to proteasome inhibitor, MG132 (Birnie, 1988; Zhou et al., 2009) . We treated HL-60 cells with CB-5083 and analyzed differentially expressed proteins in various cellular compartments—cytoplasmic extract proteins (CERs), nuclear extract proteins (NERs), and insoluble proteins (INS) — using high resolution liquid chromatography-tandem mass spectrometry (LC-MS/MS).

**Results and Discussion**

To compare the anti-proliferative effect of CB-5083 in HL-60 cells, we used the CellTiterGlo viability assay to determine the IC _50_ value of CB-5083 for both HCT116 and HL-60, which were found to be 0.31 μM and 0.45 μM, respectively ( **Fig. 1A** ). To evaluate the effects of CB-5083 on different cellular compartments in HL-60 cells, we extracted cytoplasmic (CER), nuclear (NER), and insoluble (INS) proteins using the Thermo Fisher Nuclear and Cytoplasmic Extraction kit. Proteomic analysis revealed significant differences in protein expression across these compartments compared to the DMSO control ( **Fig. 1B -D** ). We used median normalization and differential expression analysis, with a log2 fold change of 1 and a p-value of 0.05 as cutoffs. In total, 212, 390, and 2,683 proteins were upregulated, while 822, 1,002, and 303 proteins were downregulated in the CER, NER, and INS compartments, respectively ( **Table S1** ). Interestingly, as shown in **Figure 1E** , p97 expression slightly increased across all three compartments following CB-5083 treatment, with the NER compartment showing the highest log2 fold change of 2.46 (p-value = 0.0018) This may reflect a feedback mechanism in which the inhibition of p97 results in its upregulation as the cell attempts to compensate for disrupted protein homeostasis. p97 has been implicated in maintaining cellular stress responses, and its increased expression could indicate a cellular effort to counteract the inhibition of its function (Gallagher et al., 2014; Kilgas & Ramadan, 2022) . Additionally, CB-5083 treatment might induce cellular stress, further triggering upregulation of p97 as part of the cell’s protective response, helping manage protein aggregation or damage. This hypothesis is consistent with p97’s well-known role in maintaining cellular proteostasis. Interestingly, signal transducer and activator of transcription 5A (STAT5A), a key protein in the JAK/STAT signaling pathway involved in various hematological malignancies and solid tumors (Wang et al., 2023; Maurer et al., 2018) , was upregulated in the NER compartment. Conversely, STAT5A expression was downregulated in the CER compartment, suggesting a potential translocation from the cytoplasm or membrane to the nucleus, where it may perform different functions ( **Fig. 1F** ).

Further analysis of the compartment-specific effects revealed significant variations in both upregulated and downregulated proteins across the cellular compartments. Specifically, 136, 269, and 2,520 proteins were uniquely upregulated in the CER, NER, and INS compartments, respectively, while 728, 888, and 269 proteins were uniquely downregulated. The data suggest a distinct compartment-specific response to CB-5083 treatment, with a notable upregulation of proteins in the INS compartment and a marked downregulation in the CER and NER compartments. This differential expression pattern may also reflect a redistribution of protein homeostasis: the INS compartment could be accumulating proteins due to impaired degradation or enhanced aggregation, while the CER and NER compartments may experience a reduction in protein levels, possibly reflecting disrupted cellular processes. Similar phenomena have been reported with proteasome inhibitors, where impaired protein homeostasis and aggregation are common (Hirabayashi et al., 2001; Kobayashi et al., 2007) . These observations align with the role of CB-5083 in inhibiting p97 function, which is known to affect the cell’s capacity to manage stress and clear protein aggregates. However, further studies are needed to validate these mechanisms.

Using the STRING database website for protein enrichment analysis (Szklarczyk et al., 2023) , we identified distinct patterns of protein interactions, particularly involving pathways related to endoplasmic reticulum (ER) stress and immune responses. In the NER compartment, pathways associated with protein processing in the ER and various metabolic processes were significantly upregulated, while the cell cycle pathway was notably downregulated. One key protein, origin recognition complex subunit 6 (ORC6), which is critical for cell replication and has been implicated in various cancers (Prasanth et al., 2002; Lin et al., 2003) , showed a substantial reduction in the CER and NER compartments ( **Fig. 1G** ). This is consistent with our previous findings of decreased ORC6 levels following CB-5083 treatment in HCT116 cells (Wang et al., 2021) , highlighting the specific impact of CB-5083 on nuclear proteins involved in the cell cycle.

To explore the pathways associated with downregulated proteins, we compared pathway changes across the CER, NER, and INS compartments ( Fig. 1I –K). We focused on the downregulated pathways because these may be distinct from the inhibition of protein degradation, similar to proteasome inhibition as discussed previously (Wang, 2021) . Specifically, in the CER compartment, pathways related to autophagy and apoptosis were downregulated ( Fig. 1I ). In contrast, in the INS compartment, the autophagy pathway was significantly upregulated ( Fig. 1K ). In the NER compartment, cell cycle proteins represented the major enriched pathway ( Fig. 1J ). We suggest that p97 facilitates the entry of cell cycle proteins into the nucleus and protects them from degradation in the cytoplasm (Wang, 2021) . In this study, we directly demonstrated that the inhibition of p97 led to a reduced level of cell cycle proteins in the NER.

Additionally, a key protein in the autophagy pathway, ATG5, showed downregulation in the CER compartment with a log2 fold change of -2.9 and significant upregulation in the INS compartment with a log2 fold change of 6.8 ( Fig. 1H ). This contrasting expression pattern suggests a potential shift in autophagic activity within the cell, where autophagy may be suppressed in the cytoplasm (CER) but activated in regions with accumulated protein aggregates (INS). Such redistribution of ATG5 and other autophagy-related proteins could indicate a targeted cellular response to manage stress or maintain protein homeostasis in specific compartments.

Overall, the downregulated pathways in each compartment imply specific functions of p97 that are unique to the regulation of protein trafficking and transduction between the cytoplasm and the nucleus.

Our study provides a detailed proteomic analysis of the effects of a potent p97 inhibitor, CB-5083, on different cellular compartments, revealing distinct patterns of protein regulation. The protein level changes can be due to change in protein translation, degradation, and localization of in different cellular compartments. This compartmentalized effect may offer valuable insights into p97/VCP function. However, our study is limited by its focus on protein expression without functional validation of these pathways, highlighting the need for further studies to explore the underlying mechanisms and potential clinical relevance of these findings.

## Methods

**Cell Culture and anti-proliferative activity of CB-5083**

The HL-60 (ATCC, cat# CCL 240) and HCT116 (Cat# CCL-247) cell lines were expanded in RPMI1640 (Sigma-Aldrich， cat#R8758) 10% FBS (Sigma, cat#F0926), 1% penicillin-streptomycin (Fisher, cat#15140122) medium, cultured at 37 ^o^ C in 5% CO _2_ incubator. Anti-proliferative activity was measured using a CellTiter Glo® Luminescent Cell Viability Assay (Promega G7572) according to the manufacturer’s procedure. RPMI1640 containing 5% FBS and 1% Penicillin-Streptomycin was used as cell viability assay medium. 30 μL of 25 cells/μL cell suspension was plated in a 384-well plate (Greiner 781080) and CB-5083 was added, final concentration of 10.5 μM to 0.014 μM, and the plate was incubated for an additional 48 h. Cell viability was measured by adding CellTiter Glo reagent and reading the signal with BioTek Synergy Neo2 plate reader. The IC _50_ values were calculated from 4 replicates using the percentage of growth of treated cells versus the DMSO control. The results were analyzed using GraphPad Prism 10.

For proteomics analysis, cells were grown in 10 cm dishes with density 5 x 10 ^6^ / dish and treated with DMSO or 1uM CB-5083 in RPMI1640 with 5% FBS, 1% P/S medium for 24 hours in triplicate. When harvesting, cells were detached with TrypLE™ Express Enzyme (Gibco, cat# 12-604-021), washed with PBS (MilliporeSigma, cat# D8537) and spun down to collect cell pellets. Cellular viability of HL-60 treated with 1 μM CB-5083 after 24h is about 50% live cells and we collected live cells for proteomics analysis.

**Sample preparation and Liquid chromatography-tandem mass spectrometry**

We used the NE-PER Nuclear and Cytoplasmic Extraction Reagents (cat# 78835, Thermo) to isolate cytoplasmic (CER), nuclear (NER) and the insoluble pellet (INS). To each of the CER, NER samples, we used cold acetone to precipitated proteins and resuspend all pellet in 5% SDS in TEAB buffer containing 10 mM TCEP/25 mM CAA and incubated at 95 ^o^ C for 10 min and precipitated proteins to remove excel TCEP/CAA. To digest proteins, digestion mixture (100mM TEAB in 0.4% DDM, 1μg/μL trypsin, 0.5μg/μL LysC) was prepared. For CER, 60 μL add to 120 μg proteins; NER, 12 μL was add to 25μg NER proteins, and 12 μL was add to INS proteins and incubated at 37 ^o^ C overnight.

Samples were then vortexed for 15s, sonicated for 5min, spin 2min at high speed 25 ^o^ C. The peptide concentration was quantified using Pierce Quantitative Fluorometric Peptide Assay (Thermo Fisher cat# 23290), and diluted with Solvent A (97.8% H2O, 2% ACN, and 0.2% formic acid).

**Quantification and Statistical Analysis**

LC-MS/MS analysis of the digested peptides were performed on an EASY-nLC 1200 (Thermo Fisher Scientific) coupled to an Orbitrap Eclipse Tribrid mass spectrometer (Thermo Fisher Scientific). Peptides were separated on an Aurora UHPLC column (25 cm × 75 µm, 1.6 µm C18, AUR2-25075C18A, Ion Opticks) with a flow rate of 0.35 µL/min for a total duration of 196 min ionized at 1.6 kV in the positive ion mode. The gradient was composed of 6% solvent B (11 min), 6-25% B (124 min), 25-40% B (45 min), 40–98% B (1 min) and 98% B (15min); solvent A: 2% ACN and 0.2% FA in water; solvent B: 80% ACN and 0.2% FA. MS1 scans were acquired at the resolution of 120,000 from 375 to 2,000 m/z, AGC target 1e6, and maximum injection time 50 ms. MS2 scans were acquired in the ion trap using fast scan rate on precursors with 2-7 charge states and quadrupole isolation mode (isolation window: 0.7 m/z) with higher-energy collisional dissociation (HCD, 30%) activation type. Dynamic exclusion was set to 30 s. The temperature of ion transfer tube was 300°C and the S-lens RF level was set to 30. MS2 fragmentation spectra were searched with Proteome Discoverer SEQUEST (version 2.5; Thermo Scientific, Waltham, MA) against an *in silico* tryptic digested *Homo sapiens* database. The maximum number of missed cleavages was set to 2. Dynamic modifications were set to oxidation on methionine (M, +15.995 Da), and protein N-terminal acetylation (+42.011 Da). Carbamidomethylation on cysteine residues (C, +57.021 Da) was set as a fixed modification. The maximum parental mass error was set to 10 ppm, and the MS2 mass tolerance was set to 0.6 Da. The false discovery threshold was strictly set to 0.01 using the Percolator Node validated by q-value. The relative abundance of parental peptides was calculated by integrating the area under the curve of the MS1 peaks using the Minora LFQ node. The RAW data have been deposited to the ProteomeXchange Consortium via the PRIDE partner repository with the dataset identifier PXD056029 (Deutsch et al., 2020) .

Proteomics data were processed using the tidyproteomics package in R (version 4.2.2) for median normalization and data tidying (Jones et al., 2023) . Volcano plots were generated using GraphPad Prism 8, based on the outputs from tidyproteomics. Venn diagrams were created with BioVenn (https://www.biovenn.nl/), and pathway enrichment analysis was performed according to the results obtained from tidyproteomics.

Protein interaction networks were analyzed using the STRING database (version X, available at https://string-db.org/ ). This tool facilitated the identification and visualization of protein-protein interactions, as well as pathway enrichment analysis of the proteins identified in our proteomic study.

## Reagents

CB-5083 MedKoo Biosciences Cat# 206489

DMSO Fisher Scientific Cat# BP231-1

RPMI-1640 medium Sigma-Aldrich Cat# R8758

Fetal bovine serum (FBS) Sigma Cat# F0926

penicillin-streptomycin Fisher Cat# 15140122

Dulbecco’s PBS (DPBS) Sigma Cat# D8537-1

Water, Optima LC/MS Grade Fisher Scientific Cat# W6500

Acetonitrile (ACN), LC-MS Grade Fisher Scientific Cat# PI51101

Trifluoroacetic acid (TFA) Thermo Scientific Cat# 85,183

Triethylammonium bicarbonate buffer (TEAB) Sigma Cat# 17,902

TrypLE™ Select Enzyme (1X), no phenol red Gibco Cat# 50-591-420

LysC Wako Chemicals Cat# 125-05061

N-DODECYL B-D-MALTOSIDE (DDM) Thermo Fisher Cat # ***89903(5g)***

LC-MS grade formic acid (FA) Thermo Scientific Cat# 85,178

Pierce Quantitative Fluorometric Peptide Assay Thermo Scientific Cat# 23290

NE-PER Nuclear and Cytoplasmic Extraction Reagents Thermo Scientific Cat# 78835

Experimental Models: Cell lines HL-60 ATCC Cat# CCL 240 and HCT116 ATCC Cat# CCL-247

## Extended Data

Description: Table S1. Resource Type: Dataset. DOI: 10.22002/sfn6b-q2r37
